# Exercise Evaluation and Prescription—Second Edition

**DOI:** 10.3390/jfmk8010005

**Published:** 2022-12-23

**Authors:** Carl Foster, Cristina Cortis, Andrea Fusco

**Affiliations:** 1 Department of Exercise and Sport Science, University of Wisconsin-La Crosse, La Crosse, WI 54601, USA; 2 Department of Human Sciences, Society and Health, University of Cassino and Lazio Meridionale, 03043 Cassino, Italy

## Introduction

In the first volume of “Exercise Evaluation and Prescription” in the Journal of Functional Morphology and Kinesiology [1], we presented the case that prescribing exercise was integrally linked to a prior evaluation of exercise capacity and psychophysiological markers of exercise performance (including maximal oxygen uptake, maximal strength, blood lactate, ventilation, and the Rating of Perceived Exertion (RPE)). The link between evaluation and prescription provided a rational basis for prescribed exercise. In volume two, this link is extended to markers of the response to, and recovery from, systematic exercise training.

This extension is grounded in general adaptation syndrome (GAS), described by Hans Seyle 75 years ago [2]. In his study, there was a predictable response to any imposed stress. If the stress was too large, or too frequently applied, there was a predictably negative outcome (exhaustion). On the other hand, in the presence of a stressor that was neither too large nor too frequent, there was adaptation such that the organism could subsequently tolerate greater levels of stress. Although Seyle conceptualized GAS as a generic response to any stressor, it has become particularly associated with the adaptive response to exercise training.

One way of understanding the adaptive response to training is using the ‘training impulse’ (TRIMP) model of Banister et al. [3], who discovered that exercise training produces changes in both fitness and fatigue [4,5]. The TRIMP model was originally designed to work using training heart rate (HR) and time, but more recent studies have shown that it could work using RPE and time, resulting in the session RPE (sRPE) [6,7,8,9]. More recent studies have suggested that it could be understood using responses during the warm-up for subsequent exercise bouts [10], questionnaires [11], HR variability (HRV) [12,13,14], or training intensity distribution [15,16,17]. In any case, the process of monitoring training using any tool appears to be a useful method of evaluating the exerciser (athlete fitness rehabilitation) and optimizing exercise prescription [18].

This volume presents several papers, written from the perspective of optimizing training programs by better understanding the purpose and process of evaluating exercise capacity, either to better prescribe exercise training or to better understand the outcome of exercise training programs. A total of 30 papers are published, including twenty-five original articles, four reviews, and one case report; the papers focus on team sports (soccer, volleyball, handball, rugby, and basketball), individual sports (cycling, running, weightlifting, and Paralympic powerlifting), diseases (in overweight, obese, and postmenopausal women with type 2 diabetes mellitus (T2DM); individuals with pulmonary embolism; individuals with cardiovascular disease or its risk factors; individuals receiving β-blocker treatment; and breast cancer survivors), healthy individuals, and murine models.

Several studies were carried out in collegiate soccer players [12,13,14]. Aiming to compare accumulated workloads between starters and reserves in collegiate soccer, Jagim et al. [12] reported a greater distance covered by starters throughout the season, resulting in almost double the training load compared to reserves. The authors suggested the management of player workloads in collegiate soccer to address potential imbalances between starters and reserves throughout a season. In National Collegiate Athletics Association Division I women’s soccer, Askow and colleagues [13] showed that the sRPE-derived training load was strongly associated with Global Positioning System-derived measures of external load; these relationships were stronger during match play, with acceleration load and total distance exhibiting the strongest relationship with sRPE-derived training load. Finally, Redd et al. [14] found improvements in certain lower leg muscle tensiomyography measures in male soccer players in response to different warm-up protocols, including small-sided games and dynamic and plyometric exercises, regardless of the specific warm-up activities used.

Regarding the most used tests in soccer players and referees, using a meta-analysis, Grgic et al. [15] reviewed the effects of mental fatigue based on performance in the Yo-Yo test and Loughborough soccer passing and shooting tests. According to their results, mental fatigue negatively impacts endurance-based running performance, as well as soccer passing and shooting skills. Moreover, Romano et al. [16] evaluated the correlation between the official 6 × 40 m sprint and Yo-Yo Intermittent Recovery Level 1 tests and other field-based tests in male referees, and found that Illinois agility and hand-grip tests could represent simple and low-physical-impact tools for the repeated assessment and monitoring of referee fitness throughout the season. Examining the reliability, validity, sensitivity, and usefulness of the most commonly used field aerobic fitness tests, Bok and Foster [17] reported that the University of Montreal track test and the 30–15 intermittent fitness test were the best solutions for the prescription of long and short high-intensity interval training sessions, respectively.

Investigating the association between three different strength and plyometric exercises and force- and velocity-oriented change-of-direction performance in female handball and soccer players, Falch and colleagues [18] observed correlations between strength and change-of-direction performance, highlighting that the differences in step kinematics between fast and slow performers were mainly found in stronger athletes who were able to carry out a greater workload in a shorter time. Comparing the biomechanical parameters of drop jumps executed on rigid and sand surfaces, Giatsis et al. [19] showed that the compliance of sand decreases the efficiency of the mechanisms involved in the optimization of drop jump performance in male beach volleyball; this suggests that sand can offer injury prevention under the demand for large energy expenditure as it experiences less loading during the eccentric phase of the drop jump. Gómez-Carmona et al. [20] asserted that in semi-professional basketball players, training design must be individualized according to different variables, including physical fitness profiles. From a practical point of view, their results show that the advantages and weaknesses of each athlete can be determined in order to adapt training tasks and game systems to the skills and capabilities of the players. Daly and colleagues [21] documented ex-professional rugby players’ understanding of concussions and the analogies they use to describe concussions in order to determine the explicit and implicit pressures of playing professional rugby. The interviews highlighted that players, particularly coaches, did not fully understand the ramifications of concussive injury and other injury risks; this reveals the need to assist coaches in perceiving a concussion as a significant injury and not downplaying its seriousness in contact sports. Describing the conservative treatment of indirect structural muscle injuries, which are the more routinely found and more challenging type, Palermi et al. [22] reviewed therapeutic algorithms for muscle injuries and provided specific exercise rehabilitation for the four main muscle groups (i.e., the hamstrings, quadriceps, adductors, and soleus/gastrocnemius).

In the studies focusing on individual sports [23,24,25,26,27], Leo et al. [23] investigated the effects of COVID-19 restrictions on training and performance physiology measures in U23 elite cyclists, concluding that COVID-19 restrictions did not negatively affect training characteristics and physiological performance measures for a period of <30 days. Boullosa and colleagues [24] monitored the associations between HRV, training load, and performance in a middle-aged recreational female runner during a competitive 20-week macrocycle divided into a first and second mesocycle, in which her best performances over 10 km and 21 km were recorded. Their findings highlighted the practicality of concurrent HRV and sRPE monitoring in recreational runners, with the root mean square of the successive differences in R-R intervals:R-R intervals ratio indicative of specific adaptations.

Sandau et al. [25] examined the predictive validity of a new approach to the estimate one-repetition maximum (1RM) snatch computed from the two-point snatch pull force–velocity relationship, to determine the actual 1RM snatch performance in elite weightlifters. Aidar and colleagues [26] found that the maximum isometric force, rate of force development, time, velocity, and dynamic time had lower values, especially in the initial and intermediate phases in the sticking region, in Paralympic powerlifting athletes. In a subsequent study by the same research group [27] hemodynamic responses in Paralympic bench press powerlifting athletes were analyzed with respect to conventional powerlifting before and up to 60 minutes after training. According to the results of the heart pressure product, there is no risk of hemodynamic overload in conventional or Paralympic powerlifters. Moreover, training promoted a moderate hypotensive effect, with blood pressure adaptation immediately and 60 min after exercise.

Evaluating the effects of an intensive exercise program on health-related outcomes and cardio-metabolic health measures in a group of overweight and obese adults with and without T2DM, Pippi and colleagues [28] found that physical activity level and sitting time did not seem to influence the beneficial effects of exercise intervention. Bentes et al. [29] examined the influence of 12 months of vitamin D supplementation on the components of physical fitness in postmenopausal women with T2DM. Their findings showed that vitamin D supplementation alone was effective in increasing serum vitamin D levels, altering muscle strength, improving muscle function, and preventing and controlling fragility caused by T2DM and aging.

Stavrou et al. [30] assessed the effect of 8 weeks of pulmonary rehabilitation in patients with pulmonary embolism, during unsupervised and supervised pulmonary rehabilitation, on cardiopulmonary exercise testing parameters, sleep quality, quality of life, and cardiac biomarkers. Their findings highlighted that pulmonary rehabilitation may present a safe method of intervention regardless of supervision. Schultz et al. [31] aimed to document the training load accomplished by patients with known cardiovascular disease, or with risk factors likely to cause cardiovascular disease, in a community-based exercise program using both steps/day and the sRPE approach. They concluded that patients in a community clinical exercise program achieve the American College of Sports Medicine guidelines, but accomplish fewer steps than recommended. The same research group [32] developed equations for predicting the peak oxygen uptake (VO_2_peak) and ventilatory threshold (VT) during a 6 min walk test, on the basis of walking performance and terminal RPE, in clinically stable patients who took part in a cardiac rehabilitation program. The addition of terminal RPE to the 6 min walk test distance improved the prediction of maximal metabolic equivalents (METs) and METs at VT, which may have practical applications for exercise prescription.

Birnbaumer and colleagues [33] showed that the HR performance curve pattern during incremental cycle ergometer exercise was different in individuals receiving β-blocker treatment compared to that in healthy individuals, suggesting the percentage of maximum power output as a better indicator for exercise intensity prescription in this population. Di Blasio et al. [34] compared the effects of weekly personal feedback, based on objectively measured physical activity, on the trends of both daily sedentary time and physical activity in breast cancer survivors with those of an intervention also including online supervised physical exercise sessions during Italy’s COVID-19 lockdown. Their findings showed that the use of an activity tracker and its accompanying app, with the reception of weekly tailored advice and supervised online physical exercise sessions, can elicit proper physical activity in breast cancer survivors in the COVID-19 era.

Rogers et al. [35] investigated whether the anaerobic threshold derived from gas exchange is associated with the transition from a correlated to an uncorrelated random HRV pattern. HR associated with a Detrended Fluctuation Analysis alpha 1 value of 0.5 was closely related to HR at the second VT derived from gas exchange analysis, and has the potential to be a noninvasive marker for training intensity distribution and performance status. Moreover, De Blauw et al. [36] reported similar improvements in cardiovascular function, body composition, and fitness in HRV-guided high-intensity functional training when compared to predetermined high-intensity functional training, despite fewer days at high intensity.

Tyrrell et al. [37] tested a recently developed generalized model [38] to downregulate absolute training intensity in order to account for cardiovascular drift and achieve the desired internal training load using METs during exercise testing and training. Comparing acute responses to three time-matched exercise regimens consisting of sprint interval training, high-intensity interval training, and vigorous continuous training, Benítez-Flores and colleagues [39] suggested that high-intensity interval training and vigorous continuous training accumulate the longest duration at near maximal intensities, which is considered a key factor in enhancing maximal oxygen uptake (VO_2_max).

Mayer et al. [40] assessed the surface electromyographic activity of the lumbar extensor muscles during full-range-of-motion, dynamic exercise on a home back extension exercise device at three exercise loads. They concluded that dynamic exercise on a home back extension exercise device is safe and provides a mechanism to progressively activate the lumbar extensor muscles. O’ Brien et al. [41] reviewed the literature on the efficacy of flywheel inertia training to increase hamstring strength, and provided general recommendations on valuable flywheel inertia training determinants when integrating this kind of training into a hamstring strengthening program. Finally, Cariati et al. [42] showed that a well-designed aerobic training protocol in terms of speed and speed variation significantly contributes to improving synaptic plasticity and hippocampal ultrastructure, optimizing its benefits in the brains of young murine models.

Given the great success of the first and second editions of this Special Issue, we have launched a third edition, for which we hope to receive contributions focusing on the use of laboratory or field evaluations to generate training advice in patients, healthy people, and athletes.

## References

[1] Foster C., Cortis C., Fusco A. (2021). Exercise Evaluation and Prescription. J. Funct. Morphol. Kinesiol..

[2] Selye H. (1946). THhe General Adaptation Syndrome and the Diseases of Adaptation. J. Clin. Endocrinol. Metab..

[3] Banister E.W., Calvert T., Savage M.B.T. (1975). A Systems Model of Training for Athletic Performance. Aust. J. Sport. Med..

[4] Morton R.H., Fitz-Clarke J.R., Banister E.W. (1990). Modeling Human Performance in Running. J. Appl. Physiol..

[5] Fitz-Clarke J.R., Morton R.H., Banister E.W. (1991). Optimizing Athletic Performance by Influence Curves. J. Appl. Physiol..

[6] Foster C., Daines E., Hector L., Snyder A.C.W.R. (1996). Athletic Performance in Relation to Training Load. Wis. Med. J..

[7] Foster C., Boullosa D., McGuigan M., Fusco A., Cortis C., Arney B.E., Orton B., Dodge C., Jaime S., Radtke K. (2021). 25 Years of Session Rating of Perceived Exertion: Historical Perspective and Development. Int. J. Sports Physiol. Perform..

[8] Fusco A., Knutson C., King C., Mikat R.P., Porcari J.P., Cortis C., Foster C. (2020). Session RPE During Prolonged Exercise Training. Int. J. Sports Physiol. Perform..

[9] Fusco A., Sustercich W., Edgerton K., Cortis C., Jaime S.J., Mikat R.P., Porcari J.P., Foster C. (2020). Effect of Progressive Fatigue on Session RPE. J. Funct. Morphol. Kinesiol..

[10] Rodríguez-Marroyo J.A., Villa J.G., Pernía R., Foster C. (2017). Decrement in Professional Cyclists’ Performance After a Grand Tour. Int. J. Sports Physiol. Perform..

[11] Kellmann M., Bertollo M., Bosquet L., Brink M., Coutts A.J., Duffield R., Erlacher D., Halson S.L., Hecksteden A., Heidari J. (2018). Recovery and Performance in Sport: Consensus Statement. Int. J. Sports Physiol. Perform..

[12] Jagim A.R., Askow A.T., Carvalho V., Murphy J., Luedke J.A., Erickson J.L. (2022). Seasonal Accumulated Workloads in Collegiate Women’s Soccer: A Comparison of Starters and Reserves. J. Funct. Morphol. Kinesiol..

[13] Askow A.T., Lobato A.L., Arndts D.J., Jennings W., Kreutzer A., Erickson J.L., Esposito P.E., Oliver J.M., Foster C., Jagim A.R. (2021). Session Rating of Perceived Exertion (Srpe) Load and Training Impulse Are Strongly Correlated to Gps-Derived Measures of External Load in Ncaa Division i Women’s Soccer Athletes. J. Funct. Morphol. Kinesiol..

[14] Redd M.J., Starling-Smith T.M., Herring C.H., Stock M.S., Wells A.J., Stout J.R., Fukuda D.H. (2021). Tensiomyographic Responses to Warm-up Protocols in Collegiate Male Soccer Athletes. J. Funct. Morphol. Kinesiol..

[15] Grgic J., Mikulic I., Mikulic P. (2022). Negative Effects of Mental Fatigue on Performance in the Yo-Yo Test, Loughborough Soccer Passing and Shooting Tests: A Meta-Analysis. J. Funct. Morphol. Kinesiol..

[16] Romano V., Tuzi M., Di Gregorio A., Sacco A.M., Belviso I., Sirico F., Palermi S., Nurzynska D., Di Meglio F., Castaldo C. (2021). Correlation between Official and Common Field-Based Fitness Tests in Elite Soccer Referees. J. Funct. Morphol. Kinesiol..

[17] Bok D., Foster C. (2021). Applicability of Field Aerobic Fitness Tests in Soccer: Which One to Choose?. J. Funct. Morphol. Kinesiol..

[18] Falch H.N., Kristiansen E.L., Haugen M.E., van den Tillaar R. (2021). Association of Performance in Strength and Plyometric Tests with Change of Direction Performance in Young Female Team-Sport Athletes. J. Funct. Morphol. Kinesiol..

[19] Giatsis G., Panoutsakopoulos V., Kollias I.A. (2022). Drop Jumping on Sand Is Characterized by Lower Power, Higher Rate of Force Development and Larger Knee Joint Range of Motion. J. Funct. Morphol. Kinesiol..

[20] Gómez-Carmona C.D., Mancha-Triguero D., Pino-Ortega J., Ibáñez S.J. (2021). Exploring Physical Fitness Profile of Male and Female Semiprofessional Basketball Players through Principal Component Analysis—A Case Study. J. Funct. Morphol. Kinesiol..

[21] Daly E., White A., Blackett A.D., Ryan L. (2021). Pressure. A Qualitative Analysis of the Perception of Concussion and Injury Risk in Retired Professional Rugby Players. J. Funct. Morphol. Kinesiol..

[22] Palermi S., Massa B., Vecchiato M., Mazza F., De Blasiis P., Romano A.M., Di Salvatore M.G., Della Valle E., Tarantino D., Ruosi C. (2021). Indirect Structural Muscle Injuries of Lower Limb: Rehabilitation and Therapeutic Exercise. J. Funct. Morphol. Kinesiol..

[23] Leo P., Mujika I., Lawley J. (2022). Influence of COVID-19 Restrictions on Training and Physiological Characteristics in U23 Elite Cyclists. J. Funct. Morphol. Kinesiol..

[24] Boullosa D., Medeiros A.R., Flatt A.A., Esco M.R., Nakamura F.Y., Foster C. (2021). Relationships between Workload, Heart Rate Variability, and Performance in a Recreational Endurance Runner. J. Funct. Morphol. Kinesiol..

[25] Sandau I., Chaabene H., Granacher U. (2021). Predictive Validity of the Snatch Pull Force-Velocity Profile to Determine the Snatch One Repetition-Maximum in Male and Female Elite Weightlifters. J. Funct. Morphol. Kinesiol..

[26] Aidar F.J., Clemente F.M., de Matos D.G., Marçal A.C., de Souza R.F., Moreira O.C., de Almeida-Neto P.F., Vilaça-Alves J., Garrido N.D., Dos Santos J.L. (2021). Evaluation of Strength and Muscle Activation Indicators in Sticking Point Region of National-Level Paralympic Powerlifting Athletes. J. Funct. Morphol. Kinesiol..

[27] Aidar F.J., Paz Â.D.A., Gama D.D.M., de Souza R.F., Vieira Souza L.M., Santos J.L.D., Almeida-Neto P.F., Marçal A.C., Neves E.B., Moreira O.C. (2021). Evaluation of the Post-Training Hypotensor Effect in Paralympic and Conventional Powerlifting. J. Funct. Morphol. Kinesiol..

[28] Pippi R., Cugusi L., Bergamin M., Bini V., Fanelli C.G., Bullo V., Gobbo S., Di Blasio A. (2022). Impact of BMI, Physical Activity, and Sitting Time Levels on Health-Related Outcomes in a Group of Overweight and Obese Adults with and without Type 2 Diabetes. J. Funct. Morphol. Kinesiol..

[29] Bentes C.M., Costa P.B., Resende M., Netto C., Dias I., da Silveira A.L.B., Di Masi F., Miranda H., de Carvalho L.M., Marinheiro L. (2021). Effects of 12 Months of Vitamin d Supplementation on Physical Fitness Levels in Postmenopausal Women with Type 2 Diabetes. J. Funct. Morphol. Kinesiol..

[30] Stavrou V.T., Griziotis M., Vavougios G.D., Raptis D.G., Bardaka F., Karetsi E., Kyritsis A., Daniil Z., Tsarouhas K., Triposkiadis F. (2021). Supervised versus Unsupervised Pulmonary Rehabilitation in Patients with Pulmonary Embolism: A Valuable Alternative in Covid Era. J. Funct. Morphol. Kinesiol..

[31] Schultz K.L., Foster C., Radtke K., Bramwell S., Cortis C., Fusco A., Porcari J.P. (2021). Workload Accomplished in Phase III Cardiac Rehabilitation. J. Funct. Morphol. Kinesiol..

[32] Porcari J.P., Foster C., Cress M.L., Larson R., Lewis H., Cortis C., Doberstein S., Donahue M., Fusco A., Radtke K. (2021). Prediction of Exercise Capacity and Training Prescription from the 6-Minute Walk Test and Rating of Perceived Exertion. J. Funct. Morphol. Kinesiol..

[33] Birnbaumer P., Traninger H., Sattler M.C., Borenich A., Hofmann P. (2021). Pattern of the Heart Rate Performance Curve in Subjects with Beta-Blocker Treatment and Healthy Controls. J. Funct. Morphol. Kinesiol..

[34] Di Blasio A., Morano T., Lancia F., Viscioni G., Di Iorio A., Grossi S., Cianchetti E., Cugusi L., Gobbo S., Bergamin M. (2021). Effects of Activity Tracker-Based Counselling and Live-Web Exercise on Breast Cancer Survivors during Italy Covid-19 Lockdown. J. Funct. Morphol. Kinesiol..

[35] Rogers B., Giles D., Draper N., Mourot L., Gronwald T. (2021). Detection of the Anaerobic Threshold in Endurance Sports: Validation of a New Method Using Correlation Properties of Heart Rate Variability. J. Funct. Morphol. Kinesiol..

[36] Deblauw J.A., Drake N.B., Kurtz B.K., Crawford D.A., Carper M.J., Wakeman A., Heinrich K.M. (2021). High-Intensity Functional Training Guided by Individualized Heart Rate Variability Results in Similar Health and Fitness Improvements as Predetermined Training with Less Effort. J. Funct. Morphol. Kinesiol..

[37] Tyrrell T., Pavlock J., Bramwell S., Cortis C., Doberstein S.T., Fusco A., Porcari J.P., Foster C. (2021). Functional Translation of Exercise Responses from Exercise Testing to Exercise Training: The Test of a Model. J. Funct. Morphol. Kinesiol..

[38] Foster C., Anholm J.D., Bok D., Boullosa D., Condello G., Cortis C., Fusco A., Jaime S.J., de Koning J.J., Lucia A. (2020). Generalized Approach to Translating Exercise Tests and Prescribing Exercise. J. Funct. Morphol. Kinesiol..

[39] Benítez-Flores S., Magallanes C.A., Alberton C.L., Astorino T.A. (2021). Physiological and Psychological Responses to Three Distinct Exercise Training Regimens Performed in an Outdoor Setting: Acute and Delayed Response. J. Funct. Morphol. Kinesiol..

[40] Mayer J.M., Udermann B.E., Verna J.L. (2022). Electromyographic Analysis of the Lumbar Extensor Muscles during Dynamic Exercise on a Home Exercise Device. J. Funct. Morphol. Kinesiol..

[41] O’ Brien J., Browne D., Earls D., Lodge C. (2022). The Efficacy of Flywheel Inertia Training to Enhance Hamstring Strength. J. Funct. Morphol. Kinesiol..

[42] Cariati I., Bonanni R., Pallone G., Scimeca M., Frank C., Tancredi V., D’arcangelo G. (2021). Hippocampal Adaptations to Continuous Aerobic Training: A Functional and Ultrastructural Evaluation in a Young Murine Model. J. Funct. Morphol. Kinesiol..

